# Assisted Extraction with Cyclodextrins as a Way of Improving the Antidiabetic Activity of *Actinidia* Leaves

**DOI:** 10.3390/pharmaceutics14112473

**Published:** 2022-11-16

**Authors:** Szymon Sip, Anna Gościniak, Piotr Szulc, Jarosław Walkowiak, Judyta Cielecka-Piontek

**Affiliations:** 1 Department of Pharmacognosy, Faculty of Pharmacy, Poznań University of Medical Sciences, Rokietnicka 3, 60-806 Poznań, Poland; 2 Department of Agronomy, Poznań University of Life Sciences, Dojazd 11, 60-632 Poznań, Poland; 3 Department of Pediatric Gastroenterology and Metabolic Diseases, Poznan University of Medical Sciences, Szpitalna 27/33, 60-572 Poznań, Poland

**Keywords:** *Actinidia*, enzyme inhibition, cyclodextrin, extraction, diabetes, kiwi

## Abstract

Five varieties of *Actinidia* leaves (Geneva, Jumbo, Ken’s Red, Kijivska Hibridna, and Sentyabraskaya) were analyzed. The profiles of active compounds were determined, namely quercetin, rutin, epicatechin, chlorogenic acid, and kaempferol, in the raw material. Suspecting that the raw material might prove important in the treatment of diabetes, the authors assessed the antioxidant activity and the ability to inhibit enzymes responsible for the development of diabetes (α-glucosidase and α-amylase). As a result of the conducted analysis, the Ken’s Red variety was indicated as having the highest biological activity (DPPH IC_50_ = 0.332 ± 0.048; FRAP IC_0.5_ = 0.064 ± 0.005; α-glucosidase inhibition IC_50_ = 0.098 ± 0.007; α-amylase inhibition IC_50_ = 0.083 ± 0.004). In order to increase the efficiency of the extraction of active compounds from Ken’s Red variety leaves, cyclodextrins (α-CD, β-CD, and γ-CD) were used as extraction process enhancers. The obtained results showed a significant increase in the contents of extracted active compounds. In addition, the type of CD used enhanced the extraction of selected compounds (quercetin, kaempferol, rutin, chlorogenic acid, and epicatechin. This study shows that the application of cyclodextrin-based extraction significantly improved the leaf activity of the Ken’s Red variety (DPPH IC_50_ = 0.160 ± 0.019; FRAP IC_0.5_ = 0.008 ± 0.001; α-glucosidase inhibition IC_50_ = 0.040 ± 0.002; α-amylase inhibition IC_50_ = 0.012 ± 0.003).

## 1. Introduction

Increasing incidences of type 2 diabetes mellitus (DM2), obesity, cardiovascular disease, cancer, and other diseases of civilization have been observed. Highly processed food, sedentary lifestyles, and environmental pollution are some factors in the development of this epidemic in the 21st century [1]. At the same time, opportunities are being sought to support the treatment of diabetes with plant-based raw materials [2]. Many raw plant materials have been found to have antidiabetic potential, which has been widely reported in the literature [3]. An additional advantage of using plant raw materials is that they are rich in polyphenolic compounds and exhibit potent antioxidant activity [4]. Antioxidant activity protects β cells from oxidative stress-induced apoptosis and preserves their function [5].

Actinidia is a genus native to temperate eastern Asia [6]. Plants in this genus are mainly grown for their fruit, commonly known as ‘kiwi’. Today, kiwi berry is cultivated in several countries, including the USA, Chile, New Zealand, Australia, China, and most European countries, such as France, Belgium, Italy, the Netherlands, Switzerland, Austria, Poland, and Germany [7]. *Actinidia* fruits contain many nutrients, such as minerals, fiber, and vitamin C, which is why they are so valuable in the global fruit market [3]. However, it is not only the fruit that shows desirable properties for health. The leaves have also been used in traditional Chinese medicine for the symptomatic relief of numerous disorders, including digestive problems, dyspepsia, rheumatism, hemorrhoids, and cancer therapy [7]. They exhibit anti-inflammatory, antioxidant, antibacterial, antiviral, and anticancer properties [8]. *Actinidia* leaves have been used to treat gastrointestinal disorders, rheumatism, and stomach cancer [9]. There are a limited number of studies on the antidiabetic properties of the Actinidia genus, but the current ones have shown promising results. Ki Yoo et al. [10] demonstrated that ethyl acetate fraction from *Actinidia arguta* leaf improves hyperglycemia and glucose tolerance. In addition, it alleviated cholinergic system disorders and decreased oxidative stress in mice with streptozotocin-induced diabetes. A study by Lee et al. [10] on rats with streptozocin-induced diabetes showed that leaves of *Actinidia arguta* suppressed postprandial hyperglycemia by inhibiting α-glucosidase. Similar conclusions were drawn by Shirosaki et al. [11], who also confirmed a reduction in postprandial blood glucose levels in mice and confirmed the ability to inhibit α-amylase and α-glucosidase in vitro. *Actinidia* leaf contains about 40 different compounds, most of which are flavonoids [12]. Some of the most abundant compounds in the raw material are catechins. The raw material is also rich in kaempferol and chlorogenic acid, characterized by high antioxidant activity [13].

Studies show that the biological activities of many plant raw materials’ antioxidant, antidiabetic, or anti-inflammatory properties vary depending on the cultivar tested. [14,15]. Differences were also shown in the antidiabetic activity within a single species [16]. This relationship has also been noted for *Actinidia* fruit [6]. It can therefore be suspected that such an effect will also be observed in the leaves. So far, however, no comparison has been made between the activities of varieties within the species. Different testing varieties are desirable as differences in the profile of active compounds and in vitro activity can vary significantly to determine whether a species has health-promoting potential. In addition to selecting the most potent variety, the appropriate extraction method is essential in maximizing the raw material’s health-promoting potential. One way to increase the extraction efficiency is to add suitable substances such as cyclodextrins (CDs), cyclic oligosaccharides composed of glucose molecules linked by α-1,4-glycosidic bonds. The use of CDs in extraction increases the activity and quantity of active compounds, as confirmed by numerous studies [17,18]. As an environmentally friendly and relatively inexpensive substance, cyclodextrins can improve the extraction efficiency of hydrophilic and hydrophobic compounds. CDs have low toxicity and less potential to produce secondary contaminants than traditional solvents. However, it should be remembered that using the appropriate type of cyclodextrin is a critical parameter in maximizing the extraction efficiency. α-CDs are more suitable for smaller molecules, with the most commonly used β-CDs being those of medium- and γ-CDs for high-molecular-weight compounds [19]. In the case of such a complex matrix as plant material, the selection of a suitable cyclodextrin should be verified experimentally, as the chemical composition and activity of the raw materials varies greatly.

This study aimed to evaluate the antidiabetic activity of five *Actinidia* leaf varieties and assess the effect of the addition of α-CD, β-CD, and γ-CD to the extraction mixture. The antidiabetic activity was assessed by potential inhibition of α-glucosidase and α-amylase and the antioxidant potential. The use of cyclodextrins allowed us to assess the feasibility of developing an efficient extraction additive.

## 2. Materials and Methods

### 2.1. Plant Material

The plant material of five Actinidia varieties (leaf plates) for this research came from the Central Research Center for Cultivar Testing, Variety Assessment Experimental Station in Sulejów, Variety Assessment Experimental Station in Masłowice (φ = 51o15’, λ = 18o38’, H = 174 m above sea level). This location is characterized by an annual rainfall of 542 mm, with an average daily air temperature of 8.7 °C. The plantation was established in 2017. The plants were planted in a row spacing of 300 cm with rows every 200 cm. The soil of the plantation was characterized by a very high content of phosphorus, medium content of magnesium, and low content of potassium. During the growing season, the plants were fed three times with the compound fertilizer YaraMila Complex in a dose of 100 kg of fertilizer each. The plantation was protected against fungal diseases with Topsin M 500 SC at a dose of 1.5 l/ha, and pests with Mospilan 20 SP at a dose of 0.2 kg/ha.

### 2.2. Reagents and Chemicals

Standards of the determined substances: acarbose, chlorogenic acid, epicatechin, quercetin, kaempferol, and rutin, were obtained from Sigma-Aldrich (St. Louis, MO, USA).

The reagents used in the conducted studies are as follows: α-D-glucopyranoside (pNPG), α-glucosidase, acarbose, 2,2-Diphenyl-1-picrylhydrazyl, TPTZ (2,4,6-tripyridyl-S-triazine) and Iron (III) chloride hexahydrate (FeCl_3_x6H_2_O), Folin–Ciocalteu’s phenol reagent, sodium carbonate, 2,4,6-tris(2-pyridyl)-1,3,5-triazine (TPTZ, C_18_H_12_N_6_), iron(III) chloride hexahydrate (FeCl_3_·6H_2_O), Trolox, α-cyclodextrin (α-CD), β-cyclodextrin (β-CD), and γ-cyclodextrin (γ-CD) were supplied by Sigma-Aldrich, St. Louis, MO, USA. Methanol, isopropanol, and acetone (Super Purity Solvent, Methanol 215 SPS) were supplied by ROMIL Ltd., Cambridge, England.

High-quality pure and ultra-high-quality pure water were prepared using a Direct-Q 3 UV Merck Millipore (Burlington, MA, USA) purification system.

### 2.3. Extract Preparation

#### 2.3.1. Primary Extract from the Raw Material

Before the extracts were obtained from the *Actinidia* leaves, the leaves were ground in a mill (2 times) using MMK-02M (MPM, Milanówek, Poland). Then, the raw material prepared this way was sieved through a 1 mm sieve to eliminate uncrushed leaf fragments. The dried and powdered leaves of individual Actinidia cultivars were extracted with methanol at a concentration of 70%. The raw material to solvent ratio was 1 g of raw material per 50 mL of solvent. Extraction was performed in an ultrasonic bath (Ultrasonix cleaner proclean 10.0S, Ulsonix, Zielona Gora, Poland) at 40 °C for 60 min. The extractions were filtered under reduced pressure to obtain the supernatant used for the analysis. The extract prepared this way was divided into small portions and frozen at −20 °C to maintain the quantitative and qualitative composition throughout this research.

#### 2.3.2. Extraction Assisted by Cyclodextrins

Before the extracts were obtained from the *Actinidia* leaves, the leaves were ground in a mill (2 times) using MMK-02M (MPM, Milanówek, Poland). Then, the raw material prepared this way was sieved through a 1 mm sieve to eliminate uncrushed leaf fragments. Dried and powdered leaves of Ken’s Red variety and α-CD, β-CD, and γ-CD were extracted. The extracts were made in the proportion of 1 g of raw material per 1 g of individual CDs in 50 mL of 70% methanol. Extraction was performed in an ultrasonic bath used in Section 2.3.1 at 40 °C for 60 min. Later, the extracts were filtered under reduced pressure, frozen at −24 °C, and subjected to lyophilization. The process was carried out for 96 h to remove all solvents from the system; the automatic procedure was programmed in a freeze dryer (Heto PowerDry 3000, Thermo Scientific, Waltham, MA, USA). The resulting formulation was ground in an agate mortar to obtain a homogeneous powder. Dry CD extract diluted in 70% methanol in 50 mg per 1 mL was used for further analysis.

### 2.4. Determination of Total Phenolic Content (TPC)

TPC was determined using the Folin–Ciocalteu method with minor modifications. A 50 µL plant extract solution diluted 20 times was mixed with 50 µL of Folin–Ciocalteu reagent (F.-C.) and 100 µL of distilled water. The mixture was pre-incubated for 5 min at 37 °C with shaking at 100 rpm. Then, 100 µL of 20% Na_2_CO_3_ aq. solution was added and incubated for 30 min at 37 °C with shaking at 100 rpm. The absorbance was read at 750 nm against the blank sample (water instead of the extract) in sixplicate (Multiskan GO 1510, Thermo Fisher Scientific, Vantaa, Finland). TPC was expressed as mg of gallic acid equivalent per g of dry leaf mass utilizing a standard curve of gallic acid (y = 9.80847x − 0.2828; R^2^ = 0.9983) in the concentration range 0.06–0.2 mg/mL [20]. The content of TPC in the tested extract was calculated following the standard curve for gallic acid. The curve used to calculate the TPC content in the form of gallic acid as a conversion factor is presented in the Supplementary Materials Figure S1.

### 2.5. Chromatographic Determination

A selective HPLC (high-performance liquid chromatography) coupled with a DAD detector (diod-array detector) method was developed and validated for the qualitative and quantitative analysis of the obtained extracts, allowing the identification of chlorogenic acid, rutin, kaempferol, quercetin, and epicatechin. The determinations were carried out using UHPLC Nexera (Shimadzu, Kioto, Japan). This method was developed using the ReproSil Saphir 100 C18 column (250 × 4.6 mm; 5 µm) (Dr.Maisch, Ammerbuch, Germany). This method was based on gradient elution (A-0.1% trifluoroacetic acid; B-Acetonitrile), where 0 min, B-10%; 45 min, B-40%; 55 min, B-40%; 55.01 min, B-10%. During the analysis, the column was thermostated at 30 °C; detection was carried out at a wavelength of 254 nm. A 10 uL sample was injected into the column; the phase flow was set at 1.0 mL/min; the analysis lasted 60 min. Validation data for each of the standards and an exemplary chromatograph are included in the Supplementary Materials Table S1 and Figure S2.

### 2.6. Antioxidant Action

#### 2.6.1. 2,2-Diphenyl-1-picrylhydrazyl (DPPH) Assay

The DPPH assay was carried out according to Studzińska-Sroka et al. with modifications [21]. Briefly, 25.0 μL of the aqueous extracts of *Actinidia* leaves was dissolved in distilled water at different concentrations and was mixed with 175.0 μL of DPPH (Sigma-Aldrich, St. Louis, MO, USA) solution (3.9 mg in 50 mL of methanol). The reaction mixture was shaken and incubated in the dark at room temperature for 30 min. The control contained 25.0 μL of distilled water and 175.0 μL of DPPH solution. Absorbance was measured at 517 nm on the same device mentioned in Section 2.4. The inhibition of the DPPH radical by the sample was calculated according to the following formula:

DPPH scavenging activity (%) = (A0 − A1)/A0 × 100%

where:

A0—the absorbance of the control;
A1—the absorbance of the sample.

#### 2.6.2. Ferric-Reducing Antioxidant Power Assay (FRAP) Assay

According to Tiveron et al., FRAP was performed with some modifications [22]. The stock solutions of FRAP reagent included 300 mM acetate buffer (pH 3.6), 10 mM TPTZ solution in 40 mM HCl, and 20 mM FeCl_3_·6H_2_O solution. The working FRAP solution was prepared by mixing 25.0 mL of acetate buffer, 2.5 mL of TPTZ solution, and 2.5 mL of FeCl_3_·6H_2_O solution and then warmed at 37 °C before use. Briefly, 25.0 μL of the tested extracts dissolved in distilled water at different concentrations (0.2–1.0 mg/mL) were mixed with 175.0 μL of FRAP solution, shaken, and incubated at 37 °C for 30 min in dark conditions. Then, the absorbance was read at 593 nm on the same device mentioned in Section 2.4. The results were expressed as IC_0.5_, corresponding to the extract concentration required to produce a 0.5 optical density (O.D.) value.

### 2.7. Inhibition of In Vitro Activity of Enzymes

#### 2.7.1. Inhibition of α-Glucosidase

A modified spectrophotometric method developed by Telagari et al. was used to determine the inhibition of α-glucosidase by the *Acnitidia* extracts [23]. Briefly, 50.0 μL of sample solution or acarbose (positive control, 1.0–5.0 mg mL^−1^) in different concentrations, 50.0 μL of 0.1 M phosphate buffer (pH 6.8), and 30.0 µL α-glucosidase solution (1.0 U mL^−1^) were pre-incubated in 96-well plates at 37 °C for 15 min. Next, 20.0 μL of 5 mM p-nitrophenyl-α-D-glucopyranoside solution in a 0.1 M phosphate buffer (pH 6.8) was added and incubated at 37 °C for 20 min. The reaction was terminated by adding 100.0 µL of sodium carbonate (0.2 M) into the mixture. The absorbance of the liberated p-nitrophenol was measured at 405 nm (Multiskan GO 1510, Thermo Fisher Scientific, Vantaa, Finland). The absorbance of the enzyme solution without plant extracts/acarbose served as a control with total enzyme activity. The absorbance in the absence of the enzyme was used as the blind control. The enzyme inhibition rate, expressed as a percentage of inhibition, was calculated using the following formula:

% inhibition activity =((AC−AS)/AC)∗100

where:

AC—the absorbance of the control (100% enzyme activity);
AS—the absorbance of the tested sample (leaves extract or acarbose).

Three independent experiments were carried out for the investigated substances, and the average from *n* = 8 measurements was calculated. Results were expressed as means ± S.D.

#### 2.7.2. Inhibition of α-Amylase

The amylase inhibition test was performed using a spectrophotometric method. In total, 20 µL of α-amylase (2.0 U/mL) and 20 µL of the test extract were preincubated in a 96-well plate at 37 °C. After 20 min, 20 µL of 0.5% starch solution prepared in warm 0.1 M phosphate buffer (pH 6.9) was added to the wells and incubated for 20 min at 37 °C. Then, 60 µL of color reagent containing 96 mM 3,5-dinitrosalicylic acid solution (20 mL), 5.31 M potassium sodium tartrate solution in 2 M sodium hydroxide (8 mL), and deionized water (12 mL) were added to each well. The plate was incubated at 85 °C for 45 min, then cooled to room temperature, and 80 µL of water was added. The absorbance was measured at 540 nm (Multiskan GO 1510, Thermo Fisher Scientific, Vantaa, Finland). The absorbance in the presence of enzymes without extracts was used as a control for the total enzyme activity. The absorbance in the absence of enzyme was used as a blank control. The degree of enzyme inhibition was expressed as a percentage of inhibition and calculated according to the following formula:

% inhibition activity =((AC−AS)/AC)∗100

where

AC—the absorbance of the control (100% enzyme activity);
AS—the absorbance of the tested sample (leaves extract or acarbose).

Three independent experiments were carried out for the investigated substances, and the average from *n* = 8 measurements was calculated. Results were expressed as means ± S.D.

### 2.8. Statistical Analysis 

Analysis of the biological activity in vitro was performed in at least eight replicates. Statistical analysis was performed using Statistica 13.3 software (TIBCO Software Inc., Palo Alto, CA, USA). The Shapiro–Wilk test was implemented to check the data distribution normality. Statistical significance was performed using a one-way analysis of variance (ANOVA) followed by the Tukey’s HSD test test. Measurements were considered significant at *p <* 0.05.

## 3. Results and Discussion

The primary raw material from *Acnitidia* shrubs is its fruits, which are rich in polyphenols and vitamin C [24]. However, it should be noted that in most cases, secondary metabolites with biological activity in plants are stored in all parts. Earlier, a few studies showed the potential for the presence of biologically active compounds in the leaves of *Acnitidia*; thus, in the course of the conducted research, five selected varieties of *Actinidia* arguta were analyzed: Geneva (USA), Jumbo (Italy), Ken’s Red (New Zealand), Kijivska Hibridna (Ukraine), and Sentyabraskaya (Ukraine). The cultivars selected for this study have not previously been tested for antidiabetic activity and potential therapeutic application. This research aimed to determine the antioxidant potential as the fundamental parameter expected from plant materials. The production of free radicals, and thus the generated oxidative stress, contributes to the emergence and course of many chronic diseases. Thus, the ability of the plant material to sweep them guarantees a broad spectrum of activity and the possibility of therapeutic use in a wide range of diseases [25]. In addition, in vitro analyses were performed to assess the ability to inhibit enzymes responsible for the absorption of simple sugars, thus checking the usefulness of the raw material in adjunctive diabetes therapy [26,27].

The prepared extracts were analyzed regarding the content of active compounds, starting with the TPC analysis as the sum of polyphenolic compounds, which is a good indicator of the quality of the obtained raw material that then translates into the observed biological activity. Then, the contents of selected biologically active compounds in all tested varieties were determined using the HPLC method developed for this research. The selected compounds served as an analytical marker to predict the raw material’s action profile due to the targeted biological activity. The selected active compounds showed a high health-promoting potential. Thus, only selected analyses of activity were carried out to confirm the action profile of the plant’s raw material. 

### 3.1. Analysis of the Content of Active Compounds

The high contents of rutin, chlorogenic acid, and epicatechin indicate the potential for use in diabetes [28,29,30]. All indicated compounds show the potential to inhibit α-glucosidase and α-amylase, which are responsible for the breakdown of carbohydrates into simple sugars. The potential to inhibit digestive enzymes responsible for the breakdown of complex sugars translates into the possibility of using the raw material in supporting the treatment of type 2 diabetes, particularly in the early stages of the disease. This prevents a rapid increase in postprandial blood sugar [26,31].

**Figure 1:**
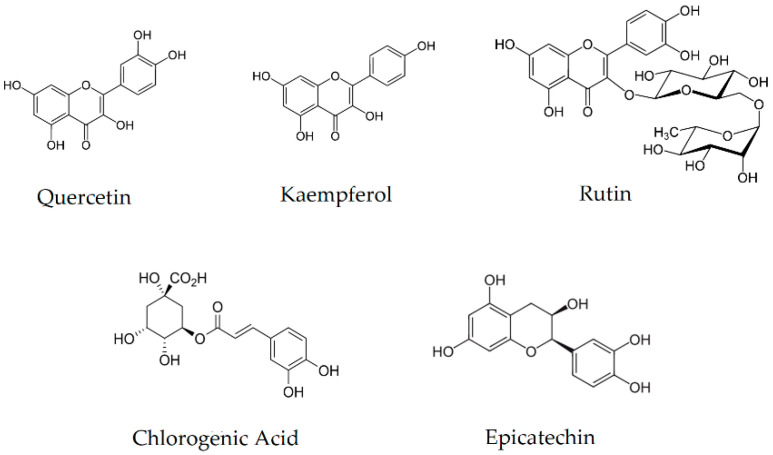
Chemical structures of the five compounds determined in the raw material.

Moreover, all marked active compounds (Figure 1) are characterized by high antioxidant activity, ensuring a complementary therapeutic effect in the ability to scavenge free radicals that generate oxidative stress, which intensifies the course of many chronic diseases [32,33,34]. In addition, they exhibit anti-inflammatory properties that are extremely important in the course and development of DM2 [35,36]. The gradual development of inflammation caused by the increased oxidative stress translates into the development of a cascade of side effects [37]. Inhibition of inflammation in the body will translate into a milder course of the disease and reduce the development of comorbidities. Additionally, kaempferol reduces the risk of cardiovascular disease, a common side effect observed in the course of DM2 [38].

The analysis of TPC allows for the straightforward evaluation of the raw material. However, it should be remembered that the simplicity of this method may generate some errors resulting from the determination of non-polyphenol compounds. The results obtained as a result of this analysis show a high correlation between the antioxidant and biological activity, allowing for the possibility of characterizing the obtained extracts [39,40]. The content of active compounds and TPC are shown in Table 1. Among the cultivars studied, Ken’s Red of New Zealand origin stands out, with the highest content of TPC (44.23 ± 0.21 mg/g dry matter (DM)), rutin (2.447 ± 0.054 mg/g DM), chlorogenic acid (2.558 ± 0.043 mg/g DM), and epicatechin (8.179 ± 0.067 mg/g DM). The second exciting variety is Sentyabraskaya of Ukrainian origin, characterized by the highest quercetin (2.144 ± 0.026 mg/g DM) but devoid of chlorogenic acid and has the lowest content of TPC (13.34 ± 0.09 mg/g DM) among the studied varieties. Despite the high content of quercetin, the lack of the indicated phenolic acid will potentially translate into a reduction in activity against sugar-digesting enzymes due to their level of enzyme inhibition. Moreover, the low content of TPC will potentially also translate into a reduction in the activity of enzymes and antioxidant activity since a close correlation has been repeatedly demonstrated between the sum of phenolic compounds and the final activity of the tested plant material [16,41].

### 3.2. Antioxidant Activity

Oxidative stress is essential in the development of many chronic diseases, such as DM2. The formed free radicals drive the formation of comorbidities and the final deterioration of the patient’s health [42]. Thus, it seems crucial that the plant materials potentially used in therapy, apart from their biological activity, show high antioxidant potential. Thanks to the use of plant materials in the supportive therapy of traditional pharmacotherapy, it is possible to improve patients’ health and relieve the course of the disease. 

The next stage of this research was to analyze the antioxidant activity and the ability to inhibit enzymes essential in the development and course of DM2. The extracts were analyzed in vitro using DPPH and FRAP assays, determining their ability to reduce free radicals. Next, their ability to inhibit α-glucosidase and α-amylase was analyzed, for which the IC_50_ results are given in Table 2. 

The analysis of the antioxidant activity in the DPPH assay showed the highest activity for Ken’s Red decay (IC_50_: 0.332 ± 0.048 mg DM/mL). The Jumbo and Geneva varieties were characterized by similar activity (IC_50_: 0.413 ± 0.077 and 0.497 ± 0.091 mg DM/mL, respectively). The indicated activity seems to be intertwined with the epicatechin content. However, the plant extract can exhibit an “entourage effect”—the intensification of the action through the sum of the effects of all components included in the extract. Usually, we do not observe close links between the antioxidant activity and the content of a specific active compound.

**Figure 2:**
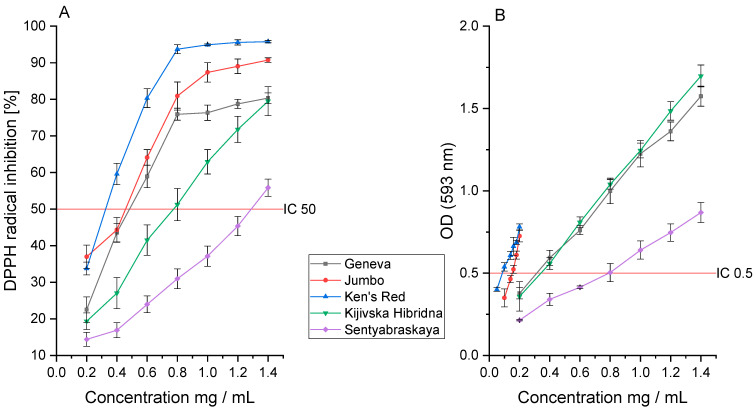
The antioxidant activity of the cultivars studied in the (**A**) DPPH and (**B**) FRAP assay.

Interestingly, the Sentyabraskaya variety was characterized by the lowest activity (IC_50_: 1.314 ± 0.098 mg DM/mL) in the tested assay, despite the high content of quercitin, which has a strong antioxidant effect. However, this indicated cultivar contains the lowest content of the sum of polyphenol compounds, which may indicate a lower activity than the other cultivars (Figure 2A). The tested extracts showed lower activity than Trolox (IC_50_: 0.092 ± 0.007 mg/mL), which was used as the reference standard. However, it should be noted that due to the technical difficulty of measuring the individual antioxidant activity of the specific substance in the extract, a lower activity of the extract in the product should be expected compared to a pure referent focused purely on the indicated antioxidant activity.

A similar analysis was carried out in the FRAP assay, confirming Ken’s Red’s highest activity (IC_0.5_: 0.064 ± 0.005 mg DM/mL). However, here, we did not observe a similar activity of the Jumbo (IC_0.5_: 0.149 ± 0.012 mg DM/mL) and Geneva (IC_0.5_: 0.320 ± 0.009 mg DM/mL) cultivars; significantly higher activity of the Jumbo cultivar was observed, similar to the Ken’s Red cultivar (Figure 2B). On the other hand, the Geneva variety was characterized by lower activity, this time similar to the Kijivska Hibridna variety (IC_0.5_: 0.333 ± 0.023 mg DM/mL). Here, we found a correlation between TPC and the activity studied, which was verified by the Pearson correlation coefficient (*p* < 0.05). The highest activity characterizes the variety with the highest content of total polyphenols. As in the case of the DPPH assay, the observed antioxidant activity is lower than that of the reference substance; however, in the FRAP assay, the Ken’s Red variety and Trolox (IC_0.5_: 0.041 ± 0.004 mg/mL) showed similar activity.

Moreover, the Sentyabraskaya variety once again showed the lowest activity (IC_0.5_: 0.753 ± 0.015 mg DM/mL) and at the same time, it is characterized by the lowest content of TPC (1.34 ± 0.09 mg/g DM), which confirms the original assumption. 

The obtained results of the antioxidant activity allow us to characterize the extracts of *Actinidia* leaves as having a high ability to scavenge free radicals. However, compared to blackberry leaves, their activity is lower [14]. Despite their activity being lower than the reference substance used, the plant extract contains a mixture of many active compounds that interact with each other, ultimately determining a more substantial biological effect in in vivo conditions [43,44]. The observed “entourage” effect guarantees activity on many levels thanks to the possibility of various mechanisms of action [45]. Due to the complementarity of the activity of many active compounds, higher activity of the plant extract is usually observed compared to the selected pure substance, which is characterized as having the highest activity [46]. 

### 3.3. Antidiabetic Activity

The next stage of this research was to estimate the biological activity of the tested extracts on the antidiabetic activity. For this purpose, an in vitro assay of α-glucosidase and α-amylase inhibition was analyzed. The proposed assays are the primary tool used for assessing the antidiabetic activity of plant materials [47]. The ability to inhibit the absorption of simple sugars is determined; further in vivo studies usually focus on evaluating changes in the intracellular metabolic pathways [48]. The ability of plant materials to inhibit enzymes responsible for the breakdown of carbohydrates into simple sugars is an excellent exponent of the raw material’s usefulness in the potential therapeutic application of DM2 treatment.

**Figure 3:**
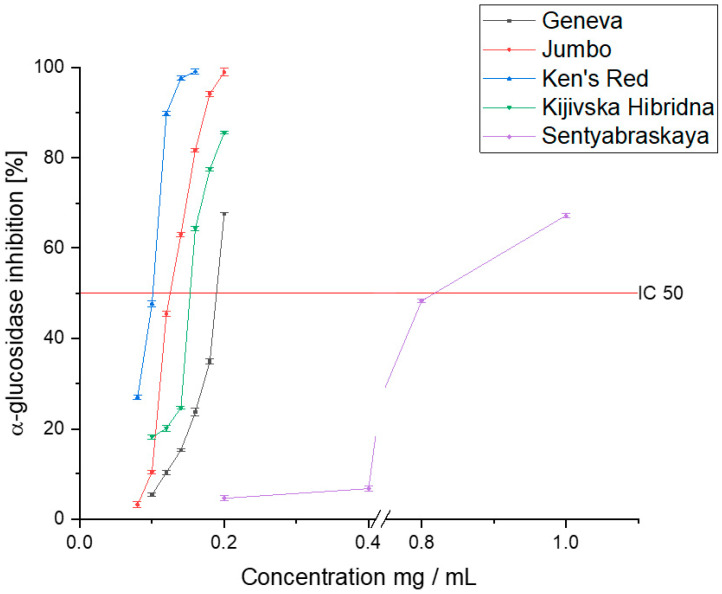
The ability of the tested cultivars to inhibit α-glucosidase.

The results indicate a high potential for inhibiting α-glucosidase (Figure 3). According to the results of the antioxidant capacity, the Ken’s Red variety showed the highest level of inhibition of the tested enzyme (IC_50_: 0.098 ± 0.007 mg DM/mL). The Geneva, Jumbo, and Kijivska Hibridna cultivars showed similar activity (0.187 ± 0.015, 0.132 ± 0.011, and 0.155 ± 0.014 mg DM/mL, respectively). However, despite the lower activity, the raw material should have a high capacity for inhibiting α-glucosidase. As a reference, the acarbose activity should be cited, which is the reference for which the determined IC_50_ value was 1.251 ± 0.071 mg/mL. The Sentyabraskaya variety, on the other hand, was characterized by a significantly lower ability to inhibit α-glucosidase (IC_50_: 0.826 ± 0.045 mg DM/mL). Despite a lower affinity for the enzyme, we can characterize all the tested varieties as showing potential to inhibit enzymes, and thus showing potential for use in diabetes therapy. Earlier studies indicated activity at a similar level of activity of the raw material [49]. Differences can be attributed to the different varieties with variable profiles of active compounds and the differences in the extraction method and the extractant used. However, there is a trend showing that the *Acnitidia* species has a potentially positive effect on alleviating the symptoms of DM2.

**Figure 4:**
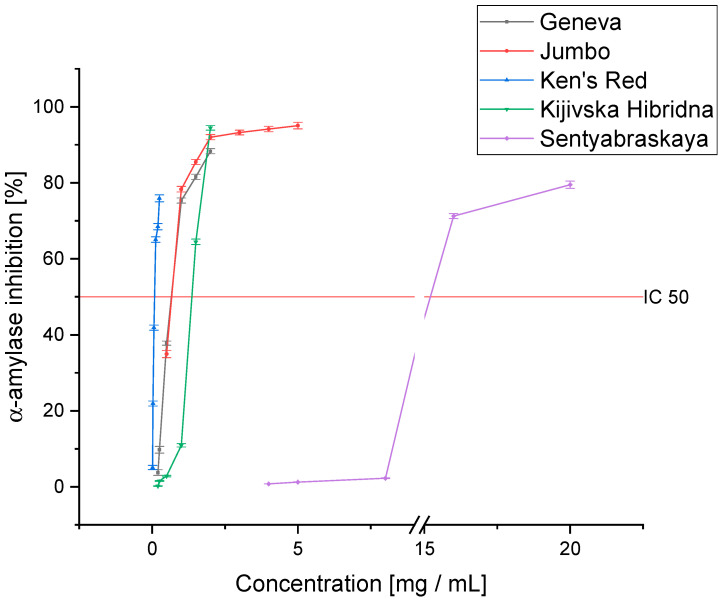
The ability of the tested cultivars to inhibit α-amylase.

In order to outline the complete profile of antidiabetic activity, an α-amylase inhibition analysis was performed (Figure 4). Again, Ken’s Red showed a significantly higher ability to inhibit the tested enzyme than the other cultivars (IC_50_: 0.083 ± 0.004 mg DM/mL). In this case, the two cultivars, Geneva and Jumbo, were characterized by similar activity (IC_50_: 0.692 ± 0.021 and 0.638 ± 0.033 mg DM/mL, respectively) while the Kijivska Hibridna cultivar was less active (IC_50_: 1.800 ± 0.066 mg DM/mL). The lowest enzyme inhibition capacity characterized the Sentyabraskaya variety (IC_50_: 14.264 ± 0.098 mg DM/mL). In this case, its activity was over 170 times lower than that of the Ken’s Red variety. This is the first analysis performed on extracts obtained from *Actinidia* leaves to determine the activity against α-amylase. However, the ability to inhibit this enzyme was confirmed by the fibers in the plant’s fruit [50]. This confirms the indicated activity of the genus. In this case, only the Ken’s Red variety showed activity higher than acarbose (IC_50_: 0.218 ± 0.01 5 mg/mL).

The results indicate a selective activity towards the tested enzymes, with a significant predominance of activity towards α-glucosidase. However, despite the lower activity against α-amylase, the raw material is characterized as having antidiabetic potential [26,51]. Plant raw materials are increasingly more often used to support the pharmacotherapy of diabetes due to the low cost of therapy and high efficiency in improving the effectiveness of pharmacotherapy [52,53]. The obtained results demonstrate that Actinidia leaves have significant potential and can be included among raw materials with high antidiabetic activity, such as *Aronia melanocarpa*, *Cornus officinalis*, *Brassica oleracea*, and *Juglans regia*. Thus, research conducted in this direction has a high application potential thanks to the possibility of further implementation of the obtained results into clinical practice. However, for a given raw material to be fully qualified as capable of showing effective antidiabetic activity, it should undergo more advanced research based on the analysis of the impact on metabolic pathways, which will allow for a complete picture of its biological activity to be obtained [54].

Interestingly, concerning the obtained activity results, chlorogenic acid generated significant biological activity in the tested raw material, which was not observed in the Sentyabraskaya variety [55]. This variety is characterized by the lowest biological activity in the tested in vitro assays. Moreover, the observed low content of total polyphenols may play a significant role due to the complementary mechanism of action of all compounds present. Moreover, despite its high biological activity, the highest quercetin content in the Sentyabraskaya variety did not seem to affect the tested plant material and generated antioxidant and biological activity [56]. This variant may show significant activity in other activity assays as indicated in the literature; however, it does not translate into antidiabetic activity.

Ken’s Red was selected for further research. This variery was characterized by the highest content of active compounds and, most importantly, it showed significantly higher antioxidant activity and the ability to inhibit the tested enzymes among the varieties tested.

### 3.4. Cyxdoxetrins Assisted Extraction

In the follow-up experimental part, after obtaining the full spectrum of the active compound’s content and selecting the cultivar that showed the most significant potential for biological activity, pre-formulation work was carried out to investigate the effect of cyclodextrins on improving the efficiency of the extraction process. Cyclodextrins are mainly used in the development of delivery systems for selected active substances [57]. However, they are increasingly being applied due to the wide range of formulation properties of the active binding compounds in inclusion complexes. Moreover, importantly, they are safe to use and have complete biocompatibility [58].

In addition, the biological activity studies were repeated to estimate the change in the activity profile. Similar studies on the use of cyclodextrins as a selective enhancer in the extraction process have already been performed [59]. Interestingly, not only the ability of cyclodextrins to increase the sum of active compounds extracted from the raw material has been demonstrated but also some specificity for the given compounds, which provides many opportunities for further development [60,61]. First, more efficient extraction of active compounds can be achieved without changing the process conditions, which may be necessary for the industry.

When optimizing the assisted extraction process with CD, attention should be paid to the binding capacity of a given cyclodextrin to selected active compounds and the strength of such an affinity. This affinity will allow for the theoretical design of the process conditions and the recovery of as many compounds as possible from the plant raw material. This theoretical approach will also potentially steer the process towards the obtainment of only a selected active substance or a group of compounds with a similar ability to bind to a given CD.

Attention should be paid to the increased cost of the extraction process using CD as extraction enhancers. However, a significantly higher yield of active compounds may translate into lower costs due to the use of less plant raw material and organic solvent, which will ensure lower waste generation and is in line with the current green industry trend. In addition, working with a smaller volume will further reduce costs due to lower media consumption. Thus, despite the increased cost of the extractor input, the final financial balance of the CD application may be positive; however, this will depend on the scale of the process and the extraction technology used.

### 3.5. Analysis of the Content of Active Compounds in Cyxdoxetrin Assisted Extraction 

First, a re-analysis of the composition of the obtained systems based on cyclodextrins was performed (Table 3). The results were converted into the content of active compounds in 1.0 g of plant material, allowing the obtained results to be compared with the original standardization. Thanks to the use of cyclodextrins, it was possible to increase the number of phenolic compounds from 44.23 ± 0.21 to 114.05 ± 0.04 mg/g DM thanks to the use of γ-CD. In the remaining applied CDs, an increase in the sum of phenolic compounds by at least 2-fold was also observed. As shown earlier, TPC correlates with the antioxidant and biological activity. Thus, we expected significantly higher activity. A significant increase in the number of active compounds in the obtained pre-formulations may result from the active binding of active compounds contained in the extract through the CD cavity [62,63]. Such binding of a given active compound will reduce the amount of this compound in the solution, allowing an additional amount to be extracted from the raw material that would generally be impossible due to saturation of the extract [64,65]. However, to fully understand the underlying mechanism of action, further studies should be carried out, focusing on the ability of CD to bind selected active compounds and, thus, the formation of inclusion complexes.

Further HPLC analysis confirmed the general tendency of cyclodextrins to improve the extraction process and the specific extraction capacity depending on the cyclodextrin used. α-CD showed the lowest specificity for the extraction of specific compounds. We observed an increase in all active compounds to a similar degree. However, it showed tremendous potential for rutin extraction. β-CD showed significant potential for the extraction of epicatechin (23.358 ± 0.078 mg/g DM) and chlorogenic acid (23.353 ± 0.031 mg/g DM). In the latter case, we observed an over 10-fold increase in the amount compared to the pure extract.

Conversely, γ-CD showed high specificity for epicatechin (21.76 ± 0.064 mg/g DM). Due to the complexity of the plant extract, we cannot clearly define the relationships that exist; however, we can conclude that some trends require further investigation. Unanimously, however, we can say that cyclodextrins are excellent enhancers of the extraction process, significantly increasing the process efficiency.

The obtained relationships indicate the importance of determining selected CDs’ affinity for the extract’s active compounds. However, due to the complexity of the extract in many active compounds, it is not easy to carry out. Further studies using single active substances and a single type of CD are necessary to reveal the exact relationships. Development of the theoretical basis of the ability of active binding compounds and cyclodextrins to be intertwined with confirmation of the existing dependencies in an experimental formis required. Only when a database of the affinities between individual active compounds is developed can further analysis using plant extracts rich in active compounds be carried out.

Due to the “entourage” effect observed in the plant extracts, significant changes in the activity resulting from changes in the ratio of active compounds can be expected. In the case of the effect of the complementary activity of successive natural active compounds, the ratio of successive compounds plays a much more significant role than their specific concentration [16]. 

### 3.6. Antioxidant Activity of CD Extracts

Analogous to the previous evaluation of the activity of extracts based on five varieties of *Acnitidi* leaves, analysis of the antioxidant and biological activity was carried out to determine their use in the treatment of diabetes. Regarding the obtained results, the IC_0.5_ and IC_50_ values are presented in Table 4.

**Figure 5:**
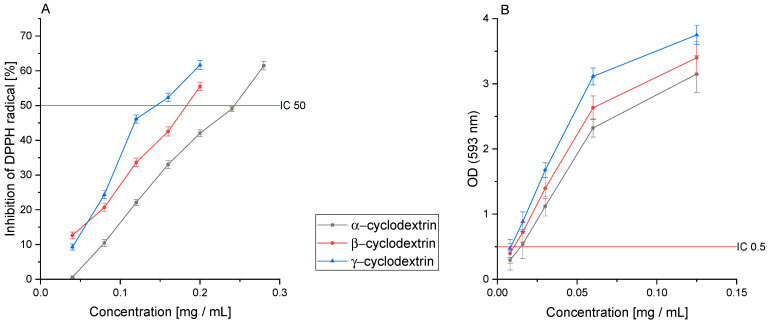
The antioxidant activity of the extracts obtained using selected cyclodextrins in the (**A**) DPPH and (**B**) FRAP assay. Significance at *p* ≤ 0.05.

In the DPPH assay, the results indicated an increase in the antioxidant potential of extracts obtained by assisted CD extraction (Figure 5A). The most significant improvement was observed using γ-CD; it was more than two times higher (IC_50_: 0.160 ± 0.019 mg DM/mL). Thus, an increase in activity leading to an improvement in the content of active compounds was confirmed. This time, thanks to the use of cyclodextrins as substances that improve the efficiency of the extraction process, the obtained IC_50_ values are close to the Trolox reference value (IC_50_: 0.092 ± 0.007 mg/mL).

Further analysis of the activity in the FRAP assay also showed an improvement in the antioxidant activity in this model. Again, γ-CD was characterized by the highest antioxidant potential (IC_0.5_: 0.008 ± 0.001 mg DM/mL), but this time, the increase in the activity was higher by as much as 8-fold. The use of α-CD and β-CD also improved the antioxidant activity by 5-fold and 6-fold, respectively (Figure 5B). Thus, the obtained results indicate a significant improvement in the raw material’s antioxidant activity through assisted CD extraction. In the case of the antioxidant activity analysis in the FRAP assay, the activity of the obtained CD extract was higher than that of Trolox (IC_0.5_: 0.041 ± 0.004 mg/mL).

The analysis of the antioxidant activity shows a large increase in the antioxidant capacity through CD as a substance that supports the extraction process. Thus, it is essential to use CD to prepare processed plant materials to improve their biological properties. However, as we can see, different CDs show different abilities to bind active compounds. Therefore, it is necessary to properly analyze a given raw material for a given activity to select the optimal CD, allowing for proper targeting and enhancement of the biological activity.

### 3.7. Antidiabetic Activity of CD Extracts

**Figure 6:**
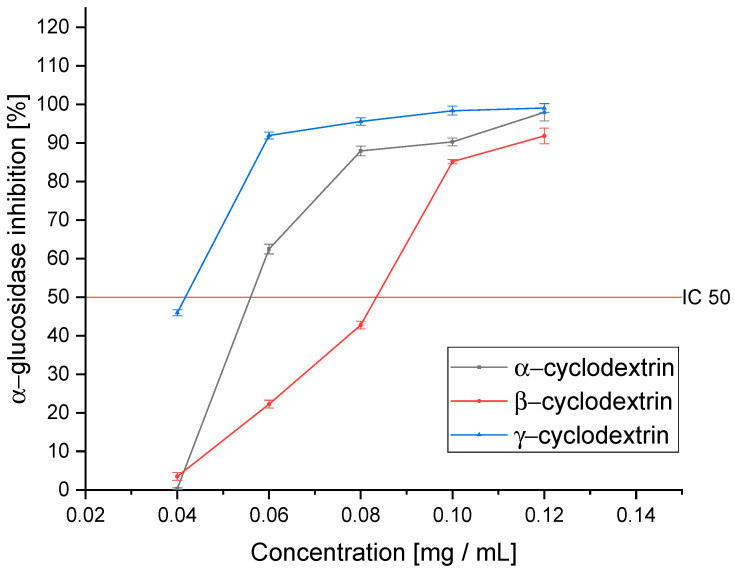
The ability of the extracts obtained using selected cyclodextrins to inhibit α-glucosidase. Significance at *p* ≤ 0.05.

Further analysis of the in vitro activity allowed us to estimate the change in the activity of the obtained cyclodextrin-based extracts (Figure 6). In all obtained systems, an improvement in the ability to inhibit α-glucosidase was observed. Again, γ-CD (IC_50_: 0.040 ± 0.002 mg DM/mL) turned out to be the best; however, in contrast to the antioxidant activity, we observed that the activity of α-CD (IC_50_: 0.059 ± 0.005 mg DM/mL) was higher than β-CD (IC_50_: 0.084 ± 0.007 mg DM/mL). The obtained differences result from the richness of the studied extract and the entourage effect, which is extremely important in the case of enzymatic inhibition analysis. The ratio between successive active compounds plays a significant role in this process. It is more important than the specific concentration due to the complementation of the mechanisms of action. In addition, it should be noted that despite the differences in activity, all of them showed a significantly higher ability to inhibit α-glucosidase than acarbose (IC_50_: 1.251 ± 0.071 mg/mL). Moreover, inhibition of the enzymes α-glucosidase and α-amylase can control glycemia and improve the response of pharmacotherapy by limiting the absorption of simple sugars [66].

**Figure 7:**
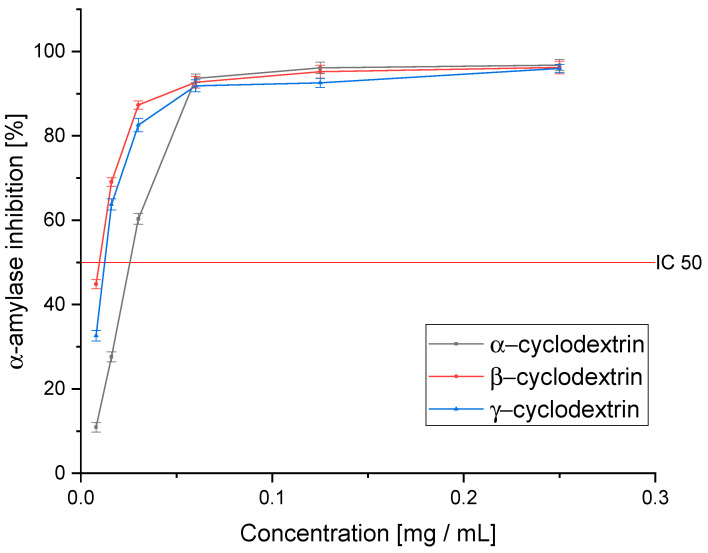
The ability of the extracts obtained using selected cyclodextrins to inhibit α-amylase. Significance at *p* ≤ 0.05.

In the tested assay of α-amylase inhibition, we observed a significantly greater improvement in activity against the enzyme than in the case of α-glucosidase (Figure 7). Interestingly, in this assay, β-CD was the best, as it improved the activity over 10-fold compared to the pure extract (IC_50_: 0.008 ± 0.002 mg DM/mL). On the other hand, γ-CD, which turned out to be the most active, also showed high activity, but the observed improvement was almost 7-fold (IC_50_: 0.012 ± 0.003 mg DM/mL). α-CD, in this case, showed only a little over a 2-fold improvement in activity (IC_50_: 0.030 ± 0.003 mg DM/mL). As in the case of the pure extract, we observed a significantly higher ability to inhibit the enzyme than acarbose (IC_50_: 0.218 ± 0.015 mg/mL).

Interestingly, in the pre-formulation, in which we observed the highest content of TPC, we also observed the highest antioxidant activity, thus confirming the usefulness of this method of raw material analysis for primary evaluation. Moreover, this relationship was also observed for α-glucosidase inhibition; however, it was not observed for α-amylase inhibition. In the latter case, the highest activity was observed for the pre-formulation containing the most epicatechin, which indicates that its concentration is related to the enzyme’s activity [67].

The first studies showed the potential of using the tested raw material in treating diabetes due to its high antioxidant activity, which provides potential protection against inflammation in an organism suffering from diabetes. Moreover, inhibition of the enzymes α-glucosidase and α-amylase can control glycemia and improve the response of pharmacotherapy by limiting the absorption of simple sugars. Moreover, the indicated activity was demonstrated in all tested cultivars, among which Ken’s Red cultivar stood out in terms of the content of active compounds and activity. Further assisted extraction showed high potential for the use of CD in such a process due to a significant improvement in the amount of extracted active compounds, which further translated into an increase in the biological activity. The studies also showed the potential of CD for selective extraction of active compounds.

## 4. Conclusions

As a result of the assisted extraction with CD, it was possible to significantly increase the contents of active compounds (quercetin, kaempferol, rutin, chlorogenic acid, and epicatechin). Further, the particular specificity of CD for the extraction of given active compounds was demonstrated, making it possible to optimize the process toward targeted extraction. Therefore, it can be concluded that the addition of cyclodextrins increases the extraction efficiency. An essential aspect of the obtained results is the possibility of translating the ability of cyclodextrins to use in the extraction process with other raw materials. Thus, another way of using cyclodextrins is identified as substances that positively influence the efficiency of the process and, with appropriate optimization, ensurer process specificity. With proper process optimization, it is possible to extract only the desired active compounds from the tested plant material.

The conducted research allowed for the most active variety to be selected, thus indicating the direction of further research, which should focus on using the most active varieties. Moreover, this research shows the potential of using *Acnitidia* leaves in adjunctive therapy for diabetes. An additional value of the research carried out is the confirmation of the high potential of using CD in the extraction process, thanks to which it is possible to conduct a more efficient extraction process using other plant material.

## Figures and Tables

**Table 1:** Quantitative analysis of the content of active compounds in the tested *Actinidia* varieties.

	Geneva [mg/g DM **]	Jumbo [mg/g DM **]	Ken’s Red [mg/g DM **]	Kijivska Hibridna [mg/g DM **]	Sentyabraskaya [mg/g DM **]
TPC	21.06 ± 0.11 ^a^	34.09 ± 0.15 ^b^	44.23 ± 0.21 ^c^	24.08 ± 0.18 ^d^	13.34 ± 0.09 ^e^
Quercetin	0.055 ± 0.002 ^a^	0.072 ± 0.004 ^b^	0.169 ± 0.010 ^c^	0.135 ± 0.009 ^d^	2.144 ± 0.026 ^e^
Kaempferol	0.135 ± 0.005 ^a^	0.122 ± 0.007 ^b^	0.086 ± 0.009 ^c^	0.124 ± 0.009 ^b^	0.373 ± 0.024 ^d^
Rutin	0.828 ± 0.009 ^a^	1.912 ± 0.027 ^b^	2.447 ± 0.054 ^c^	1.639 ± 0.027 ^d^	1.514 ± 0.022 ^e^
Chlorogenic Acid	0.066 ± 0.007 ^a^	1.553 ± 0.015 ^b^	2.558 ± 0.043 ^c^	0.383 ± 0.033 ^d^	*n*/d ***
Epicatechin	4.469 ± 0.025 ^a^	5.353 ± 0.041 ^b^	8.179 ± 0.067 ^c^	7.150 ± 0.061 ^d^	4.689 ± 0.037 ^e^

Data expressed as mean ± SD; means followed by a common letter are not significantly different by two-way ANOVA with Tukey’s post-hoc test (*p* < 0.05); ** DM—dry mass of plant material; *** *n*/d—not detected.

**Table 2:** IC_50_ and IC_0.5_ of antioxidant activity and enzyme inhibition of the studied *Actinidia* varieties.

	Antioxidant Activity [mg DM **/mL]		Ability to Inhibit Enzymes [mg DM **/mL]	
Variety	**DPPH IC_50_**	**FRAP IC_0.5_**	**α-glucosidase IC_50_**	**α-amylase IC_50_**
Geneva	0.497 ± 0.091 ^a^	0.320 ± 0.009 ^a^	0.187 ± 0.015 ^a^	0.692 ± 0.021 ^a^
Jumbo	0.413 ± 0.077 ^a^	0.149 ± 0.012 ^b^	0.132 ± 0.011 ^b^	0.638 ± 0.033 ^a^
Ken’s Red	0.332 ± 0.048 ^b^	0.064 ± 0.005 ^c^	0.098 ± 0.007 ^c^	0.083 ± 0.004 ^b^
Kijivska Hibridna	0.780 ± 0.067 ^c^	0.333 ± 0.023 ^a^	0.155 ± 0.014 ^b^	1.800 ± 0.066 ^c^
Sentyabraskaya	1.314 ± 0.098 ^d^	0.753 ± 0.015 ^d^	0.826 ± 0.045 ^d^	14.264 ± 0.098 ^d^
Reference substance	Trolox		Acarbose	
	0.092 ± 0.007	0.041 ± 0.004	1.251 ± 0.071	0.218 ± 0.015

Data expressed as mean ± SD; Means followed by a common letter are not significantly different by two-way ANOVA with Tukey’s post-hoc test (*p* < 0.05); ** DM—dry mass of plant material.

**Table 3:** Quantitative analysis of the contents of active compounds in extracts obtained using selected cyclodextrins.

	*Ken’s Red* [mg/g DM **]	α–CD [mg/g DM **]	β-CD [mg/g DM **]	γ-CD [mg/g DM **]
TPC	*41.23 ± 0.21* ^a^	93.39 ± 0.28 ^b^	82.22 ± 0.17 ^c^	114.05 ± 0.04 ^d^
Quercetin	*0.169 ± 0.010* ^a^	0.642 ± 0.016 ^b^	0.264 ± 0.015 ^c^	0.718 ± 0.021 ^d^
Kaempferol	*0.086 ± 0.009* ^a^	0.162 ± 0.011 ^b^	7.682 ± 0.033 ^c^	0.283 ± 0.025 ^d^
Rutin	*2.447 ± 0.054* ^a^	8.328 ± 0.065 ^b^	7.831 ± 0.027 ^c^	8.133 ± 0.018 ^d^
Chlorogenic Acid	*2.558 ± 0.043* ^a^	8.091 ± 0.048 ^b^	23.353 ± 0.031 ^c^	7.718 ± 0.037 ^d^
Epicatechin	*8.179 ± 0.067* ^a^	3.077 ± 0.010 ^b^	23.358 ± 0.078 ^c^	21.76 ± 0.064 ^d^

Data expressed as mean ± SD; Means followed by a common letter are not significantly different by two-way ANOVA with Tukey’s post-hoc test (*p* < 0.05); ** DM—dry mass of plant material.

**Table 4:** IC_50_ and IC_0.5_ of the antioxidant activity and enzyme inhibition of extracts obtained using selected cyclodextrins.

	Antioxidant Activity [mg DM **/mL]		Ability to Inhibit Enzymes [mg DM **/mL]	
Variety	**DPPH IC_50_**	**FRAP IC_0.5_**	**α-glucosidase IC_50_**	**α-amylase IC_50_**
*Ken’s Red*	*0.332 ± 0.048* ^a^	*0.064 ± 0.005* ^a^	*0.098 ± 0.007* ^a^	*0.083 ± 0.004* ^a^
α–CD	0.240 ± 0.031 ^b^	0.012 ± 0.001 ^b^	0.059 ± 0.005 ^b^	0.030 ± 0.003 ^b^
β-CD	0.180 ± 0.018 ^c^	0.010 ± 0.001 ^bc^	0.084 ± 0.007 ^c^	0.008 ± 0.002 ^c^
γ–CD	0.160 ± 0.019 ^c^	0.008 ± 0.001 ^c^	0.040 ± 0.002 ^d^	0.012 ± 0.003 ^c^
Reference substance	Trolox		Acarbose	
	0.092 ± 0.007	0.041 ± 0.004	1.251 ± 0.071	0.218 ± 0.015

Data expressed as mean ± SD; ** DM—dry mass of plant material; Means followed by a common letter are not significantly different by two-way ANOVA with Tukey’s post-hoc test (*p* < 0.05).

## Data Availability

The data is contained within the article or Supplementary Material.

## Supplementary Materials

The following supporting information can be downloaded at: https://www.mdpi.com/article/10.3390/pharmaceutics14112473/s1, Figure S1. The calibration curve for gallic acid used to determine the TPC content; Table S1. Validation parameters of the chromatographic method; Figure S2. Sample chromatogram for an extract.

## References

[1] Popkin B.M., Adair L.S., Ng S.W. (2012). NOW AND THEN: The Global Nutrition Transition: The Pandemic of Obesity in Developing Countries. Nutr. Rev..

[2] Kopp W. (2019). How Western Diet And Lifestyle Drive The Pandemic Of Obesity And Civilization Diseases. Diabetes Metab. Syndr. Obes..

[3] Salehi B., Ata A., Anil Kumar N., Sharopov F., Ramírez-Alarcón K., Ruiz-Ortega A., Abdulmajid Ayatollahi S., Tsouh Fokou P.V., Kobarfard F., Amiruddin Zakaria Z. (2019). Antidiabetic Potential of Medicinal Plants and Their Active Components. Biomolecules.

[4] Bors W., Michel C. (2002). Chemistry of the Antioxidant Effect of Polyphenols. Ann. New York Acad. Sci..

[5] Rajendiran D., Packirisamy S., Gunasekaran K. (2018). A Review on Role of Antioxidants in Diabetes. Asian J. Pharm. Clin. Res..

[6] Baranowska-Wójcik E., Szwajgier D. (2019). Characteristics and Pro-Health Properties of Mini Kiwi (*Actinidia Arguta*). Hortic.Environ. Biotechnol..

[7] Latocha P. (2017). The Nutritional and Health Benefits of Kiwiberry (*Actinidia Arguta*)–a Review. Plant Foods Hum. Nutr..

[8] Pinto D., Delerue-Matos C., Rodrigues F. (2020). Bioactivity, Phytochemical Profile and pro-Healthy Properties of *Actinidia Arguta*: A Review. Food Res. Int..

[9] Hong Z., Lu Y., Ran C., Tang P., Huang J., Yang Y., Duan X., Wu H. (2021). The Bioactive Ingredients in *Actinidia Chinensis* Planch. Inhibit Liver Cancer by Inducing Apoptosis. J. Ethnopharmacol..

[10] Yoo S.K., Kang J.Y., Lee U., Park S.K., Kim J.M., Han H.J., Kim D.O., Heo H.J. (2021). Improving Effect of *Actinidia Arguta* Leaf on Hyperglycemia-Induced Cognitive Dysfunction. J. Funct. Foods.

[11] SHIROSAKI M., KOYAMA T., YAZAWA K. (2008). Anti-Hyperglycemic Activity of Kiwifruit Leaf (*Actinidia Deliciosa*) in Mice. Biosci. Biotechnol. Biochem..

[12] Zhang J., Gao N., Shu C., Cheng S., Sun X., Liu C., Xin G., Li B., Tian J. (2021). Phenolics Profile and Antioxidant Activity Analysis of Kiwi Berry (*Actinidia Arguta*) Flesh and Peel Extracts From Four Regions in China. Front. Plant Sci..

[13] Almeida D., Pinto D., Santos J., Vinha A.F., Palmeira J., Ferreira H.N., Rodrigues F., Oliveira M.B.P.P. (2018). Hardy Kiwifruit Leaves (*Actinidia Arguta*): An Extraordinary Source of Value-Added Compounds for Food Industry. Food Chem..

[14] Paczkowska-Walendowska M., Gościniak A., Szymanowska D., Szwajgier D., Baranowska-Wójcik E., Szulc P., Dreczka D., Simon M., Cielecka-Piontek J. (2021). Blackberry Leaves as New Functional Food? Screening Antioxidant, Anti-Inflammatory and Microbiological Activities in Correlation with Phytochemical Analysis. Antioxidants.

[15] Insawang S., Pripdeevech P., Tanapichatsakul C., Khruengsai S., Monggoot S., Nakham T., Artrod A., D’Souza P.E., Panuwet P. (2019). Essential Oil Compositions and Antibacterial and Antioxidant Activities of Five *Lavandula Stoechas* Cultivars Grown in Thailand. Chem. Biodivers..

[16] Sip S., Szymanowska D., Chanaj-Kaczmarek J., Skalicka-Woźniak K., Budzyńska B., Wronikowska-Denysiuk O., Słowik T., Szulc P., Cielecka-Piontek J. (2022). Potential for Prebiotic Stabilized *Cornus Mas* L. Lyophilized Extract in the Prophylaxis of Diabetes Mellitus in Streptozotocin Diabetic Rats. Antioxidants.

[17] Diamanti A.C., Igoumenidis P.E., Mourtzinos I., Yannakopoulou K., Karathanos V.T. (2017). Green Extraction of Polyphenols from Whole Pomegranate Fruit Using Cyclodextrins. Food Chem..

[18] Paczkowska-Walendowska M., Szymańska E., Winnicka K., Szwajgier D., Baranowska-Wójcik E., Ruchała M.A., Simon M., Cielecka-Piontek J. (2021). Cyclodextrin as Functional Carrier in Development of Mucoadhesive Tablets Containing *Polygoni Cuspidati* Extract with Potential for Dental Applications. Pharmaceutics.

[19] Zhang S., Zhang H., Xu Z., Wu M., Xia W., Zhang W. (2017). *Chimonanthus Praecox* Extract/Cyclodextrin Inclusion Complexes: Selective Inclusion, Enhancement of Antioxidant Activity and Thermal Stability. Ind. Crops Prod..

[20] Blainski A., Lopes G.C., De Mello J.C.P. (2013). Application and Analysis of the Folin Ciocalteu Method for the Determination of the Total Phenolic Content from *Limonium Brasiliense* L. Molecules.

[21] Studzińska-Sroka E., Piotrowska H., Kucińska M., Murias M., Bylka W. (2016). Cytotoxic Activity of Physodic Acid and Acetone Extract from Hypogymnia Physodes against Breast Cancer Cell Lines. Pharm. Biol..

[22] Tiveron A.P., Melo P.S., Bergamaschi K.B., Vieira T.M.F.S., Regitano-d’Arce M.A.B., Alencar S.M. (2012). Antioxidant Activity of Brazilian Vegetables and Its Relation with Phenolic Composition. Int. J. Mol. Sci..

[23] Telagari M., Hullatti K. (2015). In-Vitro α-Amylase and α-Glucosidase Inhibitory Activity of *Adiantum Caudatum* Linn. and Celosia Argentea Linn. Extracts and Fractions. Indian J. Pharmacol..

[24] Khromykh N.O., Lykholat Y.V., Didur O.O., Sklyar T.V., Davydov V.R., Lavrentievа K.V., Lykholat T.Y. (2022). Phytochemical Profiles, Antioxidant and Antimicrobial Activity of Actinidia Polygama and A. Arguta Fruits and Leaves. Biosyst. Divers..

[25] Young I.S., Woodside J.V. (2001). Antioxidants in Health and Disease. J. Clin. Pathol..

[26] Kumar S., Narwal S., Kumar V., Prakash O. (2011). α-Glucosidase Inhibitors from Plants: A Natural Approach to Treat Diabetes. Pharmacogn. Rev..

[27] Oboh G., Isaac A.T., Akinyemi A.J., Ajani R.A. (2014). Inhibition of Key Enzymes Linked to Type 2 Diabetes and Sodium Nitroprusside Induced Lipid Peroxidation in Rats’ Pancreas by Phenolic Extracts of Avocado Pear Leaves and Fruit. Int. J. Biomed. Sci..

[28] Ghorbani A. (2017). Mechanisms of Antidiabetic Effects of Flavonoid Rutin. Biomed. Pharmacother..

[29] Abdulkhaleq L.A., Assi M.A., Noor M.H.M., Abdullah R., Saad M.Z., Taufiq-Yap Y.H. (2017). Therapeutic Uses of Epicatechin in Diabetes and Cancer. Vet. World.

[30] Yan Y., Zhou X., Guo K., Zhou F., Yang H. (2020). Use of Chlorogenic Acid against Diabetes Mellitus and Its Complications. J. Immunol. Res..

[31] Onal S., Timur S., Okutucu B., Zihnioğlu F. (2005). Inhibition of Alpha-Glucosidase by Aqueous Extracts of Some Potent Antidiabetic Medicinal Herbs. Prep. Biochem. Biotechnol..

[32] Dhanya R. (2022). Quercetin for Managing Type 2 Diabetes and Its Complications, an Insight into Multitarget Therapy. Biomed. Pharmacother..

[33] Alkhalidy H., Moore W., Wang Y., Luo J., McMillan R.P., Zhen W., Zhou K., Liu D. (2018). The Flavonoid Kaempferol Ameliorates Streptozotocin-Induced Diabetes by Suppressing Hepatic Glucose Production. Molecules.

[34] Sorriento D., De Luca N., Trimarco B., Iaccarino G. (2018). The Antioxidant Therapy: New Insights in the Treatment of Hypertension. Front. Physiol..

[35] Alam W., Khan H., Shah M.A., Cauli O., Saso L. (2020). Kaempferol as a Dietary Anti-Inflammatory Agent: Current Therapeutic Standing. Molecules.

[36] Lesjak M., Beara I., Simin N., Pintać D., Majkić T., Bekvalac K., Orčić D., Mimica-Dukić N. (2018). Antioxidant and Anti-Inflammatory Activities of Quercetin and Its Derivatives. J. Funct. Foods.

[37] Asmat U., Abad K., Ismail K. (2016). Diabetes Mellitus and Oxidative Stress—A Concise Review. Saudi Pharm. J..

[38] Ucan O., Ovayolu N. (2010). Relationship between Diabetes Mellitus, Hypertension and Obesity, and Health-Related Quality of Life in Gaziantep, a Central South-Eastern City in Turkey: The Quality of Life in Diabetes, Hypertension and Obesity. J. Clin. Nurs..

[39] Kadir D.H. (2021). Statistical Evaluation of Main Extraction Parameters in Twenty Plant Extracts for Obtaining Their Optimum Total Phenolic Content and Its Relation to Antioxidant and Antibacterial Activities. Food Sci. Nutr..

[40] Hmamou A., Eloutassi N., Alshawwa S.Z., Al kamaly O., Kara M., Bendaoud A., El-Assri E.-M., Tlemcani S., El Khomsi M., Lahkimi A. (2022). Total Phenolic Content and Antioxidant and Antimicrobial Activities of *Papaver Rhoeas* L. Organ Extracts Growing in Taounate Region, Morocco. Molecules.

[41] Xiong Y., Ng K., Zhang P., Warner R.D., Shen S., Tang H.-Y., Liang Z., Fang Z. (2020). In Vitro α-Glucosidase and α-Amylase Inhibitory Activities of Free and Bound Phenolic Extracts from the Bran and Kernel Fractions of Five Sorghum Grain Genotypes. Foods.

[42] Giacco F., Brownlee M. (2010). Oxidative Stress and Diabetic Complications. Circ. Res..

[43] Pratt D.E. (1992). Natural Antioxidants from Plant Material. Phenolic Compounds in Food and Their Effects on Health II.

[44] Eastwood M.A. (1999). Interaction of Dietary Antioxidants in Vivo: How Fruit and Vegetables Prevent Disease?. QJM: Int. J. Med..

[45] Ferber S.G., Namdar D., Hen-Shoval D., Eger G., Koltai H., Shoval G., Shbiro L., Weller A. (2020). The “Entourage Effect”: Terpenes Coupled with Cannabinoids for the Treatment of Mood Disorders and Anxiety Disorders. Curr. Neuropharmacol..

[46] Rasoanaivo P., Wright C.W., Willcox M.L., Gilbert B. (2011). Whole Plant Extracts versus Single Compounds for the Treatment of Malaria: Synergy and Positive Interactions. Malar. J..

[47] Tundis R., Loizzo M.R., Menichini F. (2010). Natural Products as α-Amylase and α-Glucosidase Inhibitors and Their Hypoglycaemic Potential in the Treatment of Diabetes: An Update. Mini Rev. Med. Chem..

[48] Si M., Lou J., Zhou C.-X., Shen J.-N., Wu H.-H., Yang B., He Q.-J., Wu H.-S. (2010). Insulin Releasing and Alpha-Glucosidase Inhibitory Activity of Ethyl Acetate Fraction of *Acorus Calamus* in Vitro and in Vivo. J. Ethnopharmacol..

[49] Lee J., Sowndhararajan K., Kim M., Kim J., Kim D., Kim S., Kim G.-Y., Kim S., Jhoo J.-W. (2014). Antioxidant, Inhibition of α-Glucosidase and Suppression of Nitric Oxide Production in LPS-Induced Murine Macrophages by Different Fractions of *Actinidia Arguta* Stem. Saudi J. Biol. Sci..

[50] Wang K., Li M., Han Q., Fu R., Ni Y. (2021). Inhibition of α-Amylase Activity by Insoluble and Soluble Dietary Fibers from Kiwifruit (*Actinidia Deliciosa*). Food Biosci..

[51] Sudha P., Zinjarde S.S., Bhargava S.Y., Kumar A.R. (2011). Potent α-Amylase Inhibitory Activity of Indian Ayurvedic Medicinal Plants. BMC Complement. Med. Ther..

[52] Schreck K., Melzig M.F. (2021). Traditionally Used Plants in the Treatment of Diabetes Mellitus: Screening for Uptake Inhibition of Glucose and Fructose in the Caco2-Cell Model. Front. Pharmacol..

[53] Kato E. (2019). Bioactive Compounds in Plant Materials for the Prevention of Diabetesand Obesity. Biosci. Biotechnol. Biochem..

[54] Fraga C.G. (2007). Plant Polyphenols: How to Translate Their in Vitro Antioxidant Actions to in Vivo Conditions. IUBMB Life.

[55] Kim J.K., Park S.U. (2019). Chlorogenic Acid and Its Role in Biological Functions: An up to Date. EXCLI J..

[56] Yang D., Wang T., Long M., Li P. (2020). Quercetin: Its Main Pharmacological Activity and Potential Application in Clinical Medicine. Oxidative Med. Cell. Longev..

[57] Loftsson T., Brewster M.E. (2010). Pharmaceutical Applications of Cyclodextrins: Basic Science and Product Development. J. Pharm. Pharmacol..

[58] Fenyvesi F., Nguyen T.L.P., Haimhoffer Á., Rusznyák Á., Vasvári G., Bácskay I., Vecsernyés M., Ignat S.-R., Dinescu S., Costache M. (2020). Cyclodextrin Complexation Improves the Solubility and Caco-2 Permeability of Chrysin. Materials.

[59] Tutunchi P., Roufegarinejad L., Hamishehkar H., Alizadeh A. (2019). Extraction of Red Beet Extract with β-Cyclodextrin-Enhanced Ultrasound Assisted Extraction: A Strategy for Enhancing the Extraction Efficacy of Bioactive Compounds and Their Stability in Food Models. Food Chem..

[60] El Darra N., Rajha H.N., Debs E., Saleh F., El-Ghazzawi I., Louka N., Maroun R.G. (2018). Comparative Study between Ethanolic and β-Cyclodextrin Assisted Extraction of Polyphenols from Peach Pomace. Int. J. Food Sci..

[61] Lakka A., Lalas S., Makris D.P. (2020). Hydroxypropyl-β-Cyclodextrin as a Green Co-Solvent in the Aqueous Extraction of Polyphenols from Waste Orange Peels. Beverages.

[62] Cid-Samamed A., Rakmai J., Mejuto J.C., Simal-Gandara J., Astray G. (2022). Cyclodextrins Inclusion Complex: Preparation Methods, Analytical Techniques and Food Industry Applications. Food Chem..

[63] Poulson B.G., Alsulami Q.A., Sharfalddin A., El Agammy E.F., Mouffouk F., Emwas A.-H., Jaremko L., Jaremko M. (2022). Cyclodextrins: Structural, Chemical, and Physical Properties, and Applications. Polysaccharides.

[64] Saokham P., Muankaew C., Jansook P., Loftsson T. (2018). Solubility of Cyclodextrins and Drug/Cyclodextrin Complexes. Molecules.

[65] Brewster M.E., Loftsson T. (2007). Cyclodextrins as Pharmaceutical Solubilizers. Adv. Drug Deliv. Rev..

[66] Kim Y., Keogh J.B., Clifton P.M. (2016). Polyphenols and Glycemic Control. Nutrients.

[67] Lim J., Kim D.K., Shin H., Hamaker B.R., Lee B.-H. (2019). Different Inhibition Properties of Catechins on the Individual Subunits of Mucosal α-Glucosidases as Measured by Partially-Purified Rat Intestinal Extract. Food Funct..

