# Supplementary material for: Assisted Extraction with Cyclodextrins as a Way of Improving the Antidiabetic Activity of Actinidia Leaves
Source: Pharmaceutics. 2022 Nov 16;14(11):2473. doi: 10.3390/pharmaceutics14112473 (PMC9695090; doi:10.3390/pharmaceutics14112473)
Supplement: Supplementary file 1 [file pharmaceutics-14-02473-s001.zip › pharmaceutics-1977782-supplementary-resubmittedv1.pdf]

pharmaceutics

MDPI

Article

Assisted Extraction with Cyclodextrins as a Way of Improving the Antidiabetic Activity of *Actinidia* Leaves

Szymon Sip [1], Anna Gościniak [1], Piotr Szulc [2], Jarosław Walkowiak [3] and Judyta Cielecka-Piontek [1,*]

[1] Department of Pharmacognosy, Faculty of Pharmacy, Poznań University of Medical Sciences, Rokietnicka 3, 60-806 Poznań, Poland
[2] Department of Agronomy, Poznań University of Life Sciences, Dojazd 11, 60-632 Poznań, Poland
[3] Department of Pediatric Gastroenterology and Metabolic Diseases, Poznan University of Medical Sciences, Szpitalna 27/33, 60-572 Poznań, Poland
* Correspondence: jpiontek@ump.edu.pl

Citation: Sip, S.; Gościniak, A.; Szulc, P.; Walkowiak, J.; Cielecka-Piontek, J. Assisted Extraction with Cyclodextrins as a Way of Improving the Antidiabetic Activity of *Actinidia* Leaves. *Pharmaceutics* **2022**, *14*, 2473. https://doi.org/10.3390/pharmaceutics14112473

Academic Editor: Maria Rosaria Lauro

Received: 2 October 2022
Accepted: 13 November 2022
Published: 16 November 2022

Publisher's Note: MDPI stays neutral with regard to jurisdictional claims in published maps and institutional affiliations.

Copyright: © 2022 by the authors. Licensee MDPI, Basel, Switzerland. This article is an open access article distributed under the terms and conditions of the Creative Commons Attribution (CC BY) license (https://creativecommons.org/licenses/by/4.0/).

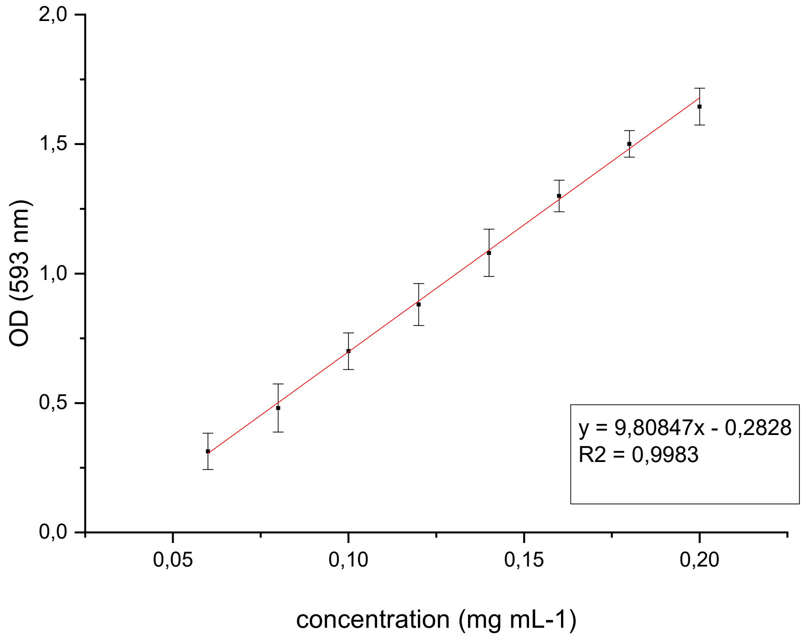

Figure S1. The calibration curve for gallic acid used to determine the TPC content

Table S1. Validation parameters of the chromatographic method

**Chlorogenic Acid**	
Calibration curve	y = 10 183 596,31x + 126 385,99
R^2	0,9988
**Rutin**	
Calibration curve	y = 20 030 315,16x + 27 676,81
R^2	0,9998
**Epicatechin**	
Calibration curve	y = 1 587 288,52x + 16 618,20
R^2	0,9991
**Kaempferol**	
Calibration curve	y = 34 905 644,67x - 20 814,12
R^2	0,9978
**Quercetin**	
Calibration curve	y = 53 673 865,16x - 30 179,19
R^2	0,9989

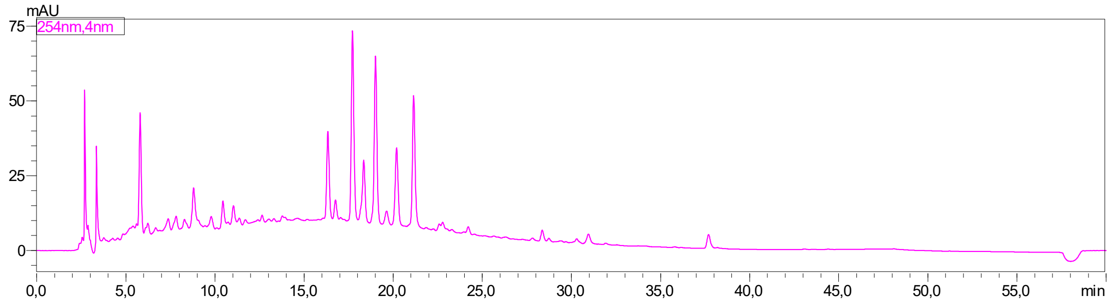

Figure S2. Sample chromatogram for an extract - reading at 254 nm.
